# Supplementary material for: Competition among Aedes aegypti larvae
Source: PLoS One. 2018 Nov 15;13(11):e0202455. doi: 10.1371/journal.pone.0202455 (PMC6237295; doi:10.1371/journal.pone.0202455)
Supplement: S1 Table — (DOCX) [file pone.0202455.s001.docx]

**S1 Table.** Sample size (number of replicates analyzed) by treatment.

| **Food level =>**  **Density (number of larvae per vial)** | **5 mg/larva** | **4 mg/larva** | **3 mg/larva** | **2 mg/larva** | **Total** |
| --- | --- | --- | --- | --- | --- |
| **4 larvae** | 5 | 4 | 4 | 2 | 15 |
| **5 larvae** | 4 | 5 | 4 | 3 | 16 |
| **6 larvae** | 5 | 5 | 5 | 4 | 19 |
| **7 larvae** | 3 | 4 | 5 | 4 | 16 |
| **8 larvae** | 4 | 5 | 5 | 5 | 19 |
| **Total** | 21 | 23 | 23 | 18 | 85 |
