# Supplementary material for: Competition among Aedes aegypti larvae
Source: PLoS One. 2018 Nov 15;13(11):e0202455. doi: 10.1371/journal.pone.0202455 (PMC6237295; doi:10.1371/journal.pone.0202455)
Supplement: S2 Table — (DOCX) [file pone.0202455.s002.docx]

**S2 Table.** Significant correlations between composite scores and variables with MANOVA significance levels and R squared by contrast.

| **Contrast (one DF for each)** | **Survival** | **Prime male mass at pupation** | **Prime male age at pupation** | **Average male mass at pupation** | **Prime female mass at pupation** | **Prime female age at pupation** | **Average female mass at pupation** | **MANOVA P<** | **R squared** |
| --- | --- | --- | --- | --- | --- | --- | --- | --- | --- |
| **FOOD LEVEL (mg per larva per vial)** |  |  |  |  |  |  |  |  |  |
| **F1: (2 mg + 3 mg) vs (4 mg + 5 mg)** |  | 0.53 |  | 0.61 | 0.77 |  | 0.71 | 0.001 | 0.92 |
| **F2: (2 mg + 4 mg) vs (3 mg + 5 mg)** |  | 0.59 | 0.28 | 0.69 | 0.69 |  | 0.64 | 0.001 | 0.86 |
| **F3: (2 mg +5 mg) vs (3 mg + 4 mg)** | 0.36 | 0.62 |  | 0.64 | 0.63 |  | 0.53 | 0.001 | 0.84 |
| **DENSITY (larvae per vial)** |  |  |  |  |  |  |  |  |  |
| **D1: 4 larvae vs 5 larvae** |  |  |  |  |  |  |  | 0.076 ns | 0.19 |
| **D2: 7 larvae vs 8 larvae** |  | 0.70 | 0.43 | 0.67 | 0.39 | 0.30 | 0.39 | 0.001 | 0.68 |
| **D3: (4 + 5 larvae) vs (7 + 8 larvae)** |  | 0.73 | 0.39 | 0.57 | 0.59 | 0.37 | 0.48 | 0.001 | 0.64 |
| **D4: 6 larvae vs (4 + 5 + 7 + 8 larvae)** |  | 0.76 | 0.33 | 0.75 | 0.57 |  | 0.52 | 0.001 | 0.60 |
| **FOOD LEVEL X DENSITY Interactions** |  |  |  |  |  |  |  |  |  |
| **F1 X D1** | 0.39 | 0.34 |  | 0.36 | 0.39 |  | 0.55 | 0.001 | 0.36 |
| **F1 X D2** |  | -0.54 | -0.54 | -0.64 | -0.48 | 0.39 | -0.37 | 0.007 | 0.27 |
| **F1 X D3** | 0.25 | 0.63 |  | 0.72 | 0.56 |  | 0.49 | 0.001 | 0.88 |
| **F1 X D4** | 0.37 | 0.52 | -0.35 | 0.53 |  |  |  | 0.018 | 0.24 |
| **F2 X D1** | 0.28 | 0.55 | 0.25 | 0.62 | 0.62 |  | 0.56 | 0.001 | 0.75 |
| **F2 X D2** |  | -0.79 | 0.27 | -0.72 | -0.30 | -0.35 | -0.30 | 0.009 | 0.26 |
| **F2 X D3** | 0.26 | 0.66 |  | 0.75 | 0.53 |  | 0.45 | 0.001 | 0.75 |
| **F2 X D4** |  |  |  |  |  |  |  | 0.814 ns | 0.05 |
| **F3 X D1** |  |  |  |  |  |  |  | 0.270 ns | 0.12 |
| **F3 X D2** |  | -0.57 | -0.61 | -0.71 | -0.40 |  | -0.40 | 0.001 | 0.32 |
| **F3 X D3** |  | -0.64 | -0.50 | -0.56 | -0.30 | -0.44 | -0.40 | 0.001 | 0.34 |
| **F3 X D4** | 0.56 | 0.40 | 0.42 | 0.39 | 0.31 |  |  | 0.003 | 0.29 |
