# Supplementary material for: Competition among Aedes aegypti larvae
Source: PLoS One. 2018 Nov 15;13(11):e0202455. doi: 10.1371/journal.pone.0202455 (PMC6237295; doi:10.1371/journal.pone.0202455)
Supplement: S3 Table — (DOCX) [file pone.0202455.s003.docx]

**S3 Table**. Mean squares, significance levels, and r squared values by single DF contrast for each of the 7 dependent variables.

| **Contrast (one DF for each)** | **Survival** | **Prime male mass at pupation** | **Prime male age at pupation** | **Average male mass at pupation** | **Prime female mass at pupation** | **Prime female age at pupation** | **Average female mass at pupation** |
| --- | --- | --- | --- | --- | --- | --- | --- |
| **FOOD LEVEL (mg per larva per vial)** |  |  |  |  |  |  |  |
| **F1: (2 mg + 3 mg) vs (4 mg + 5 mg)** |  |  |  |  |  |  |  |
| Mean squares | 1.95 | 6.70 | 16.99 | 7.51 | 42.18 | 0.29 | 36.75 |
| significance | P< .001 | P< .001 | P< .001 | P< .001 | P< .001 |  | P< .001 |
| r squared value | 0.08 | 0.17 | 0.09 | 0.21 | 0.36 |  | 0.37 |
| **F2: (2 mg + 4 mg) vs (3 mg + 5 mg)** |  |  |  |  |  |  |  |
| Mean squares | 0.23 | 4.61 | 24.13 | 5.26 | 18.98 | 76.93 | 16.60 |
| significance |  | P< .001 | P< .001 | P< .001 | P< .001 | P< .001 | P< .001 |
| r squared value |  | 0.12 | 0.12 | 0.15 | 0.16 | 0.12 | 0.17 |
| **F3: (2 mg +5 mg) vs (3 mg + 4 mg)** |  |  |  |  |  |  |  |
| Mean squares | 5.32 | 4.51 | 0.59 | 3.95 | 13.65 | 2.29 | 9.73 |
| significance | P< .001 | P< .001 |  | P< .001 | P< .001 |  | P< .001 |
| r squared value | 0.22 | 0.12 |  | 0.11 | 0.12 |  | 0.10 |
| **DENSITY (larvae per vial)** |  |  |  |  |  |  |  |
| **D1: 4 larvae vs 5 larvae** |  |  |  |  |  |  |  |
| Mean squares | 0.09 | 0.27 | 2.64 | 0.11 | 0.47 | 4.47 | 0.34 |
| significance |  | P< .01 |  | P< .05 | P< .05 |  |  |
| r squared value |  |  |  |  |  |  |  |
| **D2: 7 larvae vs 8 larvae** |  |  |  |  |  |  |  |
| Mean squares | 0.44 | 2.13 | 19.53 | 1.61 | 1.98 | 53.13 | 2.04 |
| significance |  | P< .001 | P< .001 | P< .001 | P< .001 | P< .001 | P< .001 |
| r squared value |  | 0.06 | 0.10 | 0.05 | 0.02 | 0.09 | 0.02 |
| **D3: (4 + 5 larvae) vs (7 + 8 larvae)** |  |  |  |  |  |  |  |
| Mean squares | 0.11 | 1.97 | 13.19 | 0.99 | 3.74 | 66.92 | 2.53 |
| significance |  | P< .001 | P< .001 | P< .001 | P< .001 | P< .001 | P< .001 |
| r squared value |  | 0.05 | 0.07 | 0.03 | 0.03 | 0.11 | 0.03 |
| **D4: 6 larvae vs (4 + 5 + 7 + 8 larvae)** |  |  |  |  |  |  |  |
| Mean squares | 0.16 | 1.89 | 8.25 | 1.53 | 1.53 | 0.97 | 2.67 |
| significance |  | P< .001 | P< .001 | P< .001 | P< .001 |  | P< .001 |
| r squared value |  | 0.05 | 0.04 | 0.04 | 0.01 |  | 0.03 |
| **FOOD LEVEL X DENSITY Interactions** |  |  |  |  |  |  |  |
| **F1 X D1** |  |  |  |  |  |  |  |
| Mean squares | 0.62 | 0.13 | 0.98 | 0.13 | 0.51 | 2.03 | 1.05 |
| significance | P< .05 | P< .05 |  | P< .05 | P< .05 |  | P< .001 |
| r squared value | 0.03 |  |  |  |  |  | 0.01 |
| **F1 X D2** |  |  |  |  |  |  |  |
| Mean squares | 0.04 | 0.23 | 5.32 | 0.27 | 0.52 | 15.49 | 0.33 |
| significance |  | P< .01 | P< .01 | P< .01 | P< .05 |  |  |
| r squared value |  | 0.01 | 0.03 | 0.01 | 0.01 |  |  |
| **F1 X D3** |  |  |  |  |  |  |  |
| Mean squares | 3.24 | 5.64 | 15.80 | 6.13 | 13.33 | 53.01 | 10.43 |
| significance | P< .001 | P< .001 | P< .001 | P< .001 | P< .001 | P< .001 | P< .001 |
| r squared value | 0.13 | 0.15 | 0.08 | 0.17 | 0.11 | 0.09 | 0.10 |
| **F1 X D4** |  |  |  |  |  |  |  |
| Mean squares | 0.32 | 0.17 | 1.92 | 0.15 | 0.00 | 0.11 | 0.11 |
| significance |  | P< .05 |  | P< .05 |  |  |  |
| r squared value |  |  |  |  |  |  |  |
| **F2 X D1** |  |  |  |  |  |  |  |
| Mean squares | 1.74 | 1.88 | 8.78 | 1.95 | 6.86 | 14.78 | 5.72 |
| significance | P< .001 | P< .001 | P< .001 | P< .001 | P< .001 |  | P< .001 |
| r squared value | 0.07 | 0.05 | 0.04 | 0.05 | 0.06 |  | 0.06 |
| **F2 X D2** |  |  |  |  |  |  |  |
| Mean squares | 0.00 | 0.45 | 1.27 | 0.31 | 0.20 | 11.45 | 0.20 |
| significance |  | P< .001 |  | P< .001 |  |  |  |
| r squared value |  | 0.01 |  | 0.01 |  |  |  |
| **F2 X D3** |  |  |  |  |  |  |  |
| Mean squares | 1.54 | 2.87 | 6.86 | 3.15 | 5.37 | 8.91 | 4.12 |
| significance | P< .001 | P< .001 | P< .01 | P< .001 | P< .001 |  | P< .001 |
| r squared value | 0.06 | 0.07 | 0.03 | 0.09 | 0.05 |  | 0.04 |
| **F2 X D4** |  |  |  |  |  |  |  |
| Mean squares | 0.00 | 0.00 | 1.49 | 0.00 | 0.02 | 8.21 | 0.00 |
| significance |  |  |  |  |  |  |  |
| r squared value |  |  |  |  |  |  |  |
| **F3 X D1** |  |  |  |  |  |  |  |
| Mean squares | 0.02 | 0.02 | 3.49 | 0.00 | 0.92 | 0.13 | 0.15 |
| significance |  |  | P< .05 |  |  |  |  |
| r squared value |  |  | 0.02 |  |  |  |  |
| **F3 X D2** |  |  |  |  |  |  |  |
| Mean squares | 0.13 | 0.32 | 8.78 | 0.42 | 0.48 | 1.50 | 0.49 |
| significance |  | P< .01 | P< .001 | P< .001 | P< .05 |  | P< .05 |
| r squared value |  | 0.01 | 0.04 | 0.01 |  |  |  |
| **F3 X D3** |  |  |  |  |  |  |  |
| Mean squares | 0.00 | 0.45 | 6.45 | 0.29 | 0.29 | 28.61 | 0.55 |
| significance |  | P< .001 | P< .01 | P< .001 |  | P< .05 | P< .05 |
| r squared value |  | 0.01 | 0.03 | 0.01 |  | 0.05 | 0.01 |
| **F3 X D4** |  |  |  |  |  |  |  |
| Mean squares | 0.93 | 0.14 | 3.52 | 0.11 | 0.23 | 0.00 | 0.02 |
| significance | P< .01 | P< .05 | P< .05 | P< .05 |  |  |  |
| r squared value | 0.04 |  | 0.02 |  |  |  |  |
| **Residual Mean squares (DF = 65)** | 0.11 | 0.03 | 0.75 | 0.03 | 0.09 | 4.18 | 0.10 |
| **Sum of r squared values across all contrasts** | 0.63 | 0.88 | 0.69 | 0.94 | 0.93 | 0.46 | 0.94 |
