# Supplementary material for: Competition among Aedes aegypti larvae
Source: PLoS One. 2018 Nov 15;13(11):e0202455. doi: 10.1371/journal.pone.0202455 (PMC6237295; doi:10.1371/journal.pone.0202455)
Supplement: S4 Table — (DOCX) [file pone.0202455.s004.docx]

**S4 Table.** Survival (Arcsin transformation of percent survival) by treatment.

| **Food level =>**  **Density (number of larvae per vial)** | **5 mg/larva** | **4 mg/larva** | **3 mg/larva** | **2 mg/larva** | **Mean of means [Standard Error]** |
| --- | --- | --- | --- | --- | --- |
| **4 larvae: Mean (SD)** | 1.43 (0.32) | 1.38 (0.36) | 1.21 (0.42) | 0.52 (0.00) | 1.14 [0.42] |
| **5 larvae: Mean (SD)** | 0.86 (0.14) | 1.31 (0.35) | 1.09 (0.32) | 0.83 (0.16) | 1.02 [0.22] |
| **6 larvae: Mean (SD)** | 1.24 (0.48) | 1.29 (0.40) | 0.86 (0.41) | 0.74 (0.19) | 1.03 [0.27] |
| **7 larvae: Mean (SD)** | 0.87 (0.61) | 1.30 (0.31) | 1.04 (0.32) | 0.88 (0.29) | 1.02 [0.20] |
| **8 larvae: Mean (SD)** | 0.64 (0.15) | 1.37 (0.28) | 1.43 (0.32) | 0.76 (0.24) | 1.05 [0.41] |
| **Mean of means**  **[Standard Error]** | 1.01 [0.32] | 1.33 [0.04] | 1.13 [0.21] | 0.75 [0.14] |  |
