# Supplementary material for: Competition among Aedes aegypti larvae
Source: PLoS One. 2018 Nov 15;13(11):e0202455. doi: 10.1371/journal.pone.0202455 (PMC6237295; doi:10.1371/journal.pone.0202455)
Supplement: S5 Table — (DOCX) [file pone.0202455.s005.docx]

**S5 Table.** Prime female mass at pupation (mg) by treatment.

| **Food level =>**  **Density (number of larvae per vial)** | **5 mg/larva** | **4 mg/larva** | **3 mg/larva** | **2 mg/larva** | **Mean of means [Standard Error]** |
| --- | --- | --- | --- | --- | --- |
| **4 larvae: Mean (SD)** | 4.24 (0.26) | 3.57 (0.22) | 3.20 (0.22) | 2.50 (0.40) | 3.38 [0.73] |
| **5 larvae: Mean (SD)** | 4.19 (0.22) | 3.80 (0.24) | 2.99 (0.41) | 2.52 (0.50) | 3.38 [0.76] |
| **6 larvae: Mean (SD)** | 4.41 (0.24) | 3.79 (0.29) | 3.31 (0.51) | 2.12 (0.18) | 3.41 [0.97] |
| **7 larvae: Mean (SD)** | 4.36 (0.27) | 3.78 (0.14) | 3.32 (0.42) | 2.35 (0.14) | 3.45 [0.85] |
| **8 larvae: Mean (SD)** | 4.22 (0.22) | 3.78 (0.30) | 3.18 (0.35) | 2.14 (0.26) | 3.33 [0.90] |
| **Mean of means [Standard Error]** | 4.28 [0.10] | 3.74 [0.10] | 3.20 [0.13] | 2.33 [0.19] |  |
