# Supplementary material for: Competition among Aedes aegypti larvae
Source: PLoS One. 2018 Nov 15;13(11):e0202455. doi: 10.1371/journal.pone.0202455 (PMC6237295; doi:10.1371/journal.pone.0202455)
Supplement: S6 Table — (DOCX) [file pone.0202455.s006.docx]

**S6 Table.** Average female mass at pupation (mg) by treatment.

| **Food level =>**  **Density (number of larvae per vial)** | **5 mg/larva** | **4 mg/larva** | **3 mg/larva** | **2 mg/larva** | **Mean of means [Standard Error]** |
| --- | --- | --- | --- | --- | --- |
| **4 larvae: Mean (SD)** | 4.11 (0.23) | 3.45 (0.20) | 2.95 (0.13) | 2.50 (0.40) | 3.25 [0.69] |
| **5 larvae: Mean (SD)** | 3.92 (0.35) | 3.53 (0.21) | 2.93 (0.40) | 2.52 (0.50) | 3.23 [0.62] |
| **6 larvae: Mean (SD)** | 3.81 (0.33) | 3.55 (0.32) | 3.22 (0.53) | 2.08 (0.19) | 3.17 [0.76] |
| **7 larvae: Mean (SD)** | 4.28 (0.30) | 3.50 (0.28) | 3.00 (0.30) | 2.20 (0.21) | 3.25 [0.87] |
| **8 larvae: Mean (SD)** | 4.11 (0.23) | 3.50 (0.32) | 2.92 (0.28) | 2.10 (0.26) | 3.16 [0.86] |
| **Mean of means [Standard Error]** | 4.05 [0.18] | 3.51 [0.04] | 3.00 [0.12] | 2.28 [0.21] |  |
