# Supplementary material for: Competition among Aedes aegypti larvae
Source: PLoS One. 2018 Nov 15;13(11):e0202455. doi: 10.1371/journal.pone.0202455 (PMC6237295; doi:10.1371/journal.pone.0202455)
Supplement: S7 Table — (DOCX) [file pone.0202455.s007.docx]

**S7 Table.** Prime male mass at pupation (mg) by treatment.

| **Food level =>**  **Density (number of larvae per vial)** | **5 mg/larva** | **4 mg/larva** | **3 mg/larva** | **2 mg/larva** | **Mean of means [Standard Error]** |
| --- | --- | --- | --- | --- | --- |
| **4 larvae: Mean (SD)** | 2.46 (0.11) | 2.20 (0.15) | 2.22 (0.13) | 2.07 (0.06) | 2.24 [0.16] |
| **5 larvae: Mean (SD)** | 2.55 (0.15) | 2.34 (0.30) | 2.23 (0.20) | 1.79 (0.09) | 2.23 [0.32] |
| **6 larvae: Mean (SD)** | 2.64 (0.17) | 2.51 (0.23) | 2.23 (0.19) | 1.71 (0.07) | 2.27 [0.41] |
| **7 larvae: Mean (SD)** | 2.65 (0.23) | 2.21 (0.09) | 2.26 (0.17) | 1.78 (0.07) | 2.23 [0.36] |
| **8 larvae: Mean (SD)** | 2.62 (0.10) | 2.47 (0.40) | 2.19 (0.20) | 1.97 (0.31) | 2.31 [0.29] |
| **Mean of means [Standard Error]** | 2.58 [0.08] | 2.35 [0.14] | 2.23 [0.03] | 1.86 [0.15] |  |
