# Supplementary material for: Competition among Aedes aegypti larvae
Source: PLoS One. 2018 Nov 15;13(11):e0202455. doi: 10.1371/journal.pone.0202455 (PMC6237295; doi:10.1371/journal.pone.0202455)
Supplement: S8 Table — (DOCX) [file pone.0202455.s008.docx]

**S8 Table.** Average male mass at pupation (mg) by treatment.

| **Food level =>**  **Density (number of larvae per vial)** | **5 mg/larva** | **4 mg/larva** | **3 mg/larva** | **2 mg/larva** | **Mean of means [Standard Error]** |
| --- | --- | --- | --- | --- | --- |
| **4 larvae: Mean (SD)** | 2.60 (0.27) | 2.19 (0.11) | 2.21 (0.12) | 2.07 (0.06) | 2.27 [0.23] |
| **5 larvae: Mean (SD)** | 2.57 (0.13) | 2.31 (0.27) | 2.17 (0.26) | 1.70 (0.16) | 2.19 [0.36] |
| **6 larvae: Mean (SD)** | 2.60 (0.11) | 2.42 (0.12) | 2.15 (0.16) | 1.60 (0.08) | 2.19 [0.44] |
| **7 larvae: Mean (SD)** | 2.49 (0.11) | 2.16 (0.09) | 2.19 (0.19) | 1.72 (0.05) | 2.14 [0.32] |
| **8 larvae: Mean (SD)** | 2.53 (0.13) | 2.33 (0.13) | 2.06 (0.12) | 1.84 (0.19) | 2.19 [0.30] |
| **Mean of means [Standard Error]** | 2.56 [0.05] | 2.28 [0.11] | 2.16 [0.06] | 1.79 [0.18] |  |
