# Supplementary material for: Competition among Aedes aegypti larvae
Source: PLoS One. 2018 Nov 15;13(11):e0202455. doi: 10.1371/journal.pone.0202455 (PMC6237295; doi:10.1371/journal.pone.0202455)
Supplement: S9 Table — (DOCX) [file pone.0202455.s009.docx]

**S9 Table.** Prime male age at pupation (days) by treatment.

| **Food level =>**  **Density (number of larvae per vial)** | **5 mg/larva** | **4 mg/larva** | **3 mg/larva** | **2 mg/larva** | **Mean of means [Standard Error]** |
| --- | --- | --- | --- | --- | --- |
| **4 larvae: Mean (SD)** | 5.6 (0.5) | 5.3 (0.5) | 5.5 (0.6) | 5.5 (0.7) | 5.48 [0.13] |
| **5 larvae: Mean (SD)** | 6.5 (0.6) | 5.0 (0.0) | 5.3 (0.5) | 5.7(0.6) | 5.63 [0.65] |
| **6 larvae: Mean (SD)** | 6.0 (1.0) | 5.0 (0.0) | 5.2 (0.4) | 6.0 (0.8) | 5.55 [0.53] |
| **7 larvae: Mean (SD)** | 6.3 (0.6) | 5.3 (0.5) | 5.0 (0.0) | 5.0 (0.0) | 5.40 [0.62] |
| **8 larvae: Mean (SD)** | 8.5 (3.3) | 5.2 (0.4) | 5.2 (0.4) | 5.0 (0.0) | 5.98 [1.69] |
| **Mean of means [Standard Error]** | 6.58 [1.13] | 5.16 [0.15] | 5.24 [0.18] | 5.44 [0.44] |  |
