# Supplementary material for: Competition among Aedes aegypti larvae
Source: PLoS One. 2018 Nov 15;13(11):e0202455. doi: 10.1371/journal.pone.0202455 (PMC6237295; doi:10.1371/journal.pone.0202455)
Supplement: S10 Table — (DOCX) [file pone.0202455.s010.docx]

**S10 Table.** Prime female age at pupation (days) by treatment.

| **Food level =>**  **Density (number of larvae per vial)** | **5 mg/larva** | **4 mg/larva** | **3 mg/larva** | **2 mg/larva** | **Mean of means [Standard Error]** |
| --- | --- | --- | --- | --- | --- |
| **4 larvae: Mean (SD)** | 7.4 (1.8) | 6.0 (0.8) | 7.8 (2.2) | 8.0 (2.8) | 7.30 [0.90] |
| **5 larvae: Mean (SD)** | 8.0 (2.0) | 6.2 (0.4) | 8.3 (3.2) | 7.0 (1.0) | 7.38 [0.96] |
| **6 larvae: Mean (SD)** | 7.0 (1.7) | 6.6 (0.5) | 7.2 (1.8) | 7.0 (0.0) | 6.95 [0.25] |
| **7 larvae: Mean (SD)** | 12.0 (4.6) | 6.0 (0.0) | 7.6 (2.5) | 6.5 (0.6) | 8.03 [2.73] |
| **8 larvae: Mean (SD)** | 9.5 (1.0) | 6.2 (0.4) | 9.6 (2.6) | 9.8 (4.1) | 8.78 [1.72] |
| **Mean of means [Standard Error]** | 8.78 [2.04] | 6.20 [0.24] | 8.10 [0.93] | 7.66 [1.31] |  |
