# Supplementary material for: Competition among Aedes aegypti larvae
Source: PLoS One. 2018 Nov 15;13(11):e0202455. doi: 10.1371/journal.pone.0202455 (PMC6237295; doi:10.1371/journal.pone.0202455)
Supplement: S11 Table — (DOCX) [file pone.0202455.s011.docx]

| S 11 Table. Explanation of contrasts. For the MANOVA the contrast compares all 7 dependent variables at the same time. Each ANOVA addresses a single dependent variable. This is not a mathematical description of the contrasts. | |
| --- | --- |
| Single df contrasts | **Explanation** |
| Food Level (mg per larva per vial) |  |
| F1: (2 mg + 3 mg) vs (4 mg + 5 mg) | This contrast compares the low food treatments to the high food treatments. The low food treatments are all those vials with 2 mg/food per larva or 3 mg/food per larva. The high food treatments are all those vials with 4 mg/food per larva or 5 mg/food per larva. If there is a significant difference between the means of the two groups, represented as (2 mg + 3 mg) vs (4 mg + 5 mg), then the variable is affected by the higher food level. The mean values of the variable should indicate how it is affected. |
| F2: (2 mg + 4 mg) vs (3 mg + 5 mg) | This contrast compares the lowest food level (2 mg/food per larva) plus the second highest food level (4 mg/food per larva) with the second lowest food level (3 mg/food per larva) plus the highest food level (5 mg/food per larva). If there is a significant difference between the means of the two groups, represented as (2 mg + 4 mg) vs (3 mg + 5 mg), then the variable is affected by the difference between either the two low food levels or the two high food levels, or both. The mean values of the variable should indicate how it is affected. |
| F3: (2 mg +5 mg) vs (3 mg + 4 mg) | This contrast compares the lowest food level (2 mg/food per larva) plus the highest food level (5 mg/food per larva) with the two intermediate food levels (3 mg/food per larva and 4 mg/food per larva). If there is a significant difference between the means of the two groups, represented as (2 mg + 5 mg) vs 3 mg + 4 mg), then the variable is affected differently at the high or low food level than at the middle food levels. The mean values of the variable should indicate how it is affected. |
| Density (larvae per vial) |  |
| D2: 7 larvae vs 8 larvae | This contrast compares the two highest densities with each other (7 larvae/vial with 8 larvae/vial). If there is a significant difference between the means of the two groups, then vials with 7 larvae are different from those with 8 larvae for the variable. The mean values of the variable should indicate how it is affected. |
| D3: (4 + 5 larvae) vs (7 + 8 larvae) | This contrast compares the two lowest densities with the two highest densities of larvae. The low density treatments are all those vials with 4 larvae or 5 larvae in them. The high density treatments are all those vials with 7 larvae or 8 larvae in them. If there is a significant difference between the means of the two groups, represented as (4 + 5 larvae) vs (7 + 8 larvae), then the variable is affected differently by the low density compared to high density. The mean values of the variable should indicate how it is affected. |
| D4: 6 larvae vs (4 + 5 + 7 + 8 larvae) | This contrast compares the middle density to the high and low densities combined. The middle density treatment is all those vials with 6 larvae in them. The high and low density treatments are all the other vials: 4 larvae/vial, 5 larvae/vial, 7 larvae/vial and 8 larvae/vial. If there is a significant difference between the means of the two groups, represented at 6 larvae vs (4 + 5 + 7 + 8 larvae), then the effect of adding one more larva to the vial changes depending on how many larvae are in the vial already. The mean values of the variable should indicate how it is affected. |
| Food Level X Density Interactions |  |
| F1 X D3 | This interaction crosses the low food vs high food contrast (F1 above) with the low density vs high density contrast (D3 above). If there is a significant interaction between food and density there is likely to be competition. This comparison is the easiest to interpret for competition. The mean values of the variable should indicate how it is affected. Competition is expected to be lowest in the high food, low density vials and highest in the low food, high density vials. The high food, high density vials and low food, low density vials should experience intermediate levels of competition. |
| F2 X D1 | This interaction crosses the second food contrast, which is sensitive to differences between the two low food levels, or the two high food levels, or both, and the first density contrast, between the two lowest densities. The first density contrast was not significant in itself so there is no difference between the two lowest densities as a main effect, but it is significant for this interaction contrast. The first density contrast compares the two lowest densities, 4 larvae/vial and 5 larvae/vial. This interaction means that there is a difference across the 4 food levels at the lowest densities. The mean values of the variable should indicate how it is affected. Competition is expected to be lowest in the high food, low density vials, in this case the 3 mg food/larva and 5 mg food/larvae treatments with 4 larvae/vial. Competition is expected to be highest in the low food, high density vials, in this case, the 2 mg food/larva and the 4 mg food/larva treatments with 5 larvae/vial. Competition should be intermediate in the other two treatment combinations. |
