# Supplementary material for: Competition among Aedes aegypti larvae
Source: PLoS One. 2018 Nov 15;13(11):e0202455. doi: 10.1371/journal.pone.0202455 (PMC6237295; doi:10.1371/journal.pone.0202455)
Supplement: S12 Table — (DOCX) [file pone.0202455.s012.docx]

S12 Table. Means (Standard Deviations) and N (sample size) for Prime male mass at two food levels and 9 different sex ratios (% males).

| Percent Males | 3 mg food/larva | 4 mg food/larva |
| --- | --- | --- |
|  | Mean mass in mg (SD) N | Mean mass in mg (SD) N |
| 17 | 1.63 () 1 | - () 0 |
| 20 | 1.75 (0.27) 2 | 2.02 (0.31) 2 |
| 33 | 1.95 (0.10) 2 | - () 0 |
| 40 | 1.81 (0.12) 5 | 2.20 (0.55) 3 |
| 50 | 2.36 (0.43) 3 | 2.39 (0.15) 4 |
| 60 | 2.06 (0.36) 8 | 2.36 (0.19) 5 |
| 67 | 1.98 (0.04) 2 | 2.38 (0.05) 3 |
| 80 | 2.11 (0.08) 3 | 2.49 () 1 |
| 83 | 2.05 () 1 | - () 0 |
