# Supplementary material for: Competition among Aedes aegypti larvae
Source: PLoS One. 2018 Nov 15;13(11):e0202455. doi: 10.1371/journal.pone.0202455 (PMC6237295; doi:10.1371/journal.pone.0202455)
Supplement: S1 Text — (DOCX) [file pone.0202455.s015.docx]

S1 Text. Differences between males and females—detailed analysis

The MANOVA gives us insight into the relationships among the variables. Increased food level (mg/larva) increases mass for all larvae. Females are affected more than males, Prime females more than Average females, Average males more than Prime males. Increased food level has no effect on Prime female age at pupation and only a small effect Prime male age at pupation. Prime male age at pupation increases with increasing food level.

Also, in the MANOVA, increased density (larvae/vial) increases mass for all larvae. Males are affected more than females, Prime males more than Average males, Prime females more than Average females. Increased density increases age at pupation for both Prime males and Prime females; Prime male age at pupation is affected more than Prime female age at pupation.

There are three significant interactions in the MANOVA; these interactions describe how competition and total food per vial affect the interplay of the food level and density treatments. The three main MANOVA interactions (F1 X D3, F2 X D1, and F2 X D3) affect male mass more than female mass, Average males more than Prime males, and Prime females more than Average females. The interactions have no effect on Prime female age at pupation. Only the F2 X D1 interaction affects Prime male age at pupation.

The univariate r squared values for food level and for density are similar to the MANOVA correlations for the relationships between male mass and female mass. The mean values (mg) of all four mass variables increase with increasing food level. Density does not have the same effect on each of the four mass variables. Prime male mass is highest at the highest density. Average male mass is highest at the lowest density. Prime female mass increases with density, but is lowest at the highest density. Average female mass is also lowest at the highest density. In the univariate analyses, food level alone accounts for more than half of the variation in the two female mass variables and just less than half of the variation in the two male mass variables. Density alone accounts for much less of the variation than food level in all four mass variables. The interactions between food level and density account for more of the variation in these variables than density alone.

The r squared values for Prime male and Prime female age at pupation are relatively higher than the corresponding MANOVA correlations for both the food level and density treatments. There are effects of the experimental treatment on age at pupation that did not contribute to the MANOVA significance. The Prime male and female age at pupation are both highest at the highest food level. They are also both highest at the highest density. These are the vials with the highest total food per vial. Increased mass at pupation is beneficial to the fitness of the adult mosquito of both sexes. Increased age at pupation is potentially detrimental to adult males, probably less so to adult females. Total food per vial appears to affect the larvae and some of the effect of density may be due to the total food per vial. This should be apparent in the interactions.

The r squared values for the interactions F1 X D3 and F2 X D3, correspond to the MANOVA correlations: they explain the variance in male mass more than female mass, Average males more than Prime males, and Prime females more than Average females. The r squared values for the interaction F2 X D1 (low density treatments) are slightly higher for females than for males, and explain the same amount of variance in the mass of Prime females and Average females, and in the mass of Prime males and Average males. In contrast to the MANOVA correlations, there is a significant interaction (F1 X D3) for Prime female age at pupation, and all three interactions have significant r squared values for Prime male age at pupation.

All three of the main interactions consist of 4 treatment combinations: least competition, most competition, least food per vial and most food per vial (Tables 4-7). For instance, Survival is highest in vials with the least competition and lowest in vials with the least food per vial. Both are low density treatments. If competition is affecting the mass at pupation, the mass should be highest in the vials with the least competition. This is only true for the Average male mass and the Prime and Average female mass in the F2 X D1 interaction (low density). Alternatively, competition could be causing the lowest masses to occur in the vials with the most competition. This is the case for the Prime male and the Average male in all three interactions, and the Prime and Average females in two of the interactions (F1 X D3 and F2 X D3). Males and females respond differently to the high and low competition treatment combinations and females respond differently in low densities (D1) than across the full range of densities (D3). If total food per vial is important, then the lowest masses should be in the vials with the least total food; this is only true for Prime and Average females in the F2 X D1 interaction. Alternatively, the highest masses could be in the vials with the most total food. This is true for the Prime males in all three interactions and the Prime and Average females in the F1 X D3 and F2 X D3 interactions.

Across the full range of the experimental treatments Prime males, Prime females and Average females grow largest in the vials with the most total food and smallest in the vials with the most competition. However, females at low density grow largest in vials with the least competition and smallest in vials with the least total food. Average males grow largest in the vials with the least competition and smallest in the vials with the most competition. Prime and Average females respond to the treatment conditions similarly to one another, but the Prime and Average males respond to the same treatment conditions differently from each other. Competition among males differs from competition among females. The food level and total food per vial affect competition for both sexes, but these effects are different across sexes.

Prime male age at pupation is greatest in the vials with the most food and least in the vials with the most competition. This corresponds to the Prime male mass. In the vials with the most food, the Prime male grows largest and delays pupation. In the vials with the most competition, the Prime male is smallest and pupates earliest. For the one significant interaction affecting the Prime female age at pupation, the greatest age at pupation is in the vials with the most food and the earliest is in the vials with the least competition, another difference between males and females.

Age and mass at pupation together are a measure of growth rate. For both the Prime male and the Prime female, the growth rates are highest in the vials with the least competition. This highest growth rate for the Prime female is higher than the growth rate for the Prime male in each of these interactions. Despite taking longer to pupate, females grow faster than males in the vials with least competition. [Note that growth is expected to be a sigmoid curve in which the instantaneous growth rate increases to an inflection point and then decreases towards zero. If the males pupate lower on the curve than females, then they could appear to have a lower growth rate than females even if they grow at the same rate.] The slowest growth rates for males and females are in the vials with the highest densities (most competition and most food per vial). The F2 X D1 interaction differs in that the Prime female has the lowest growth rate in the vials with the least total food.

There are consistent differences between male and female larvae across the three main interactions. Competition among males and among females needs to be considered separately. Furthermore, the results of the sex ratio experiment show that the percent of males in the vial affects the growth of males at low food levels, but not that of females; this supports the observation that there is a difference in the way the two sexes compete for the same food resource.
