# Supplementary material for: Competition among Aedes aegypti larvae
Source: PLoS One. 2018 Nov 15;13(11):e0202455. doi: 10.1371/journal.pone.0202455 (PMC6237295; doi:10.1371/journal.pone.0202455)
Supplement: S2 Text — (DOCX) [file pone.0202455.s016.docx]

S2 Text. Competition among females—detailed analysis

The mass at pupation of females is directly related to the food level. Food level explains 8-10 times the experimental variance that density explains and three times the variance that the three main interactions explain (Tables 2 and 3). In contrast, age at pupation is better explained by density than by food level or the interactions, although the Prime female age at pupation is the variable least affected by the experimental conditions. Competition is described by the interactions, so competition appears to be less important to females than the food level.

These two experiments look at competition among females in five ways: the interactions identify differences in 1) mass and 2) age at pupation under various levels of competition; 3) the difference between the Prime and the Average female masses indicates possible interference competition; 4) the differences in growth rate of the Prime female in the different treatment combinations is another measure of competition; and 5) the lack of effect of the percent of males on the mass of females indicates that females outcompete males.

1) The mass at pupation responds differently in the F2 X D1 interaction than in the other two. All the vials in this interaction have either 4 larvae or 5 larvae; this is the low density treatment in the F1 X D3 and F2 X D3 interactions. Because the food is added on a per larva basis, the total food levels in these vials are lower as well. For this interaction, the largest females are in the vials with the least competition and the smallest are in the vials with the least total food (for both Prime and Average female mass at pupation). These are the vials with 4 larvae. In the higher density treatment, the vials with 5 larvae, the masses at the high food level are lower, but the masses at the low food level are higher, than in the vials with 4 larvae. The addition of a larva plus an increment of food reduces the size of the females at the high food level, but increases the size of females at the low food level.

In the F1 X D3 and F2 X D3 interactions, the largest females are in the vials with the most total food and the smallest females are in those with the most competition; these are both the high density treatments (7 larvae or 8 larvae per vial). The masses of Prime and Average females in the low density treatments (4 larvae or 5 larvae per vial) are similar to the masses observed in the F2 X D1 interaction, suggesting that the difference is due to the higher density or higher total food level in the other vials. The masses of females in the F1 X D3 and F2 X D3 interactions are higher than that of the females in the F2 X D1 interaction in the vials with the greatest total food, and lower than those in the F2 X D1 interaction in the vials with the greatest competition. The additional larvae in the high density vials, plus the additional increments of food, increase the pupal masses of females in the high food vials and decrease the masses of females in the low food (most competition) vials. For females, competition is affected by food level (food/larva), density (larvae/vial) and total food per vial.

2) The Prime female pupates earliest in the vials with the least competition and latest in the vials with the most total food per vial. The Prime female age at pupation is less affected by competition (or any treatment) than any other variable.

3) There is no indication of interference competition based on the size distribution of females at pupation. At the lowest total food levels this distribution is compressed rather than elongated. In the other vials the difference between the Prime female and the Average female mass is approximately the same (0.20 mg). Comparing the Prime females in the vials with the lowest total food to those in the vials with the most competition (equivalent food/larva) and similarly comparing the Average females in those two treatments, it appears that the tighter distribution of sizes is due to the relative increase in size of the Average females at the lower density. Two things are likely causes: 1) the number of particles becomes limiting to the females earlier in the vials with the lowest total food and they switch to retention before they have developed the same size distribution as in the other vials, and 2) there is little or no difference in the ability of females to extract nutrients from the retained particles, so they all grow equally well from that point. When the females in the low total food vials switch to retention from filtering, the net effect is a more equal distribution of food among them, as compared to females in the vials with the most competition.

4) The growth rate of the Prime female is greatest in the vials with the least competition. Growth rate is approximated by dividing the mean values of the Prime female mass by the corresponding mean value of the Prime female age at pupation for each treatment combination. No additional significance tests were applied.

5) Female mass at pupation is unaffected by the percent of males in the vial (at the food levels and densities tested). Females larvae appear to be unaffected by competition with male larvae.

At low density the least competition results in the largest females and the least total food results in the smallest females. At high density the most total food results in the largest females and the most competition results in the smallest females. The low density treatments across all three interactions are comparable in treatment conditions and outcomes, so the high density (7 larvae or 8 larvae) is qualitatively different. Competition among females at high density results in smaller masses at pupation despite equivalent food/larva levels. High total food levels at high density result in larger masses at pupation despite equivalent food/larva levels. If total food per vial were entirely responsible for these observations the addition of a larva (from 4 larvae to 5 larvae) and an increment of food (per larva) should not lower the mass at pupation of females at the high food level (more total food) and increase the mass of females at the low food level (more competition). The 5 larvae in the vials with the most competition do better than the 4 larvae in the vials with the same food/larva, while the 5 larvae in the vials with the most food don’t do as well as the 4 larvae with the same food/larva, the vials with the least competition. At the higher densities (7 larvae or 8 larvae), the reverse is true. The 7 or 8 larvae in the vials with the most competition do worse than the 4 or 5 larvae in vials with the same food/larva, while 7 or 8 larvae in the vials with the most food do better that the 4 or 5 larvae in the vials with the least competition (equivalent food/larva).

For female larvae, there are 6 environmental conditions indicated by the three main interactions:

1) Vials with the least competition—These females grow fastest and pupate earliest. This is the benchmark to compare the other treatments against.

2) Vials with the least total food—These females pupate at a smaller mass than those in vials with the least competition and there is a tighter distribution of masses (smaller difference in size between the Prime and Average females) than in all the other vials. There is no indication of interference competition; the reduced size distribution is probably due to females retaining the particles in their guts for longer periods to extract more nutrients. In these vials, it appears that the low total food causes females to switch from filtering to retention at an earlier instar before large differences in size have developed, resulting in the compressed size distribution. Retention supplies fewer nutrients over time than filtering particles and passing them rapidly through the gut, resulting in smaller size at pupation and a slower growth rate than in the vials with the least competition.

3) Vials with the most total food (5 larvae)—These females have more total food than those in the vials with the least competition (4 larvae), but they don’t grow as fast or as large. The addition of one more larva even with the incremental food/larva reduces the growth rate and final mass. The larvae filter rapidly for long enough to develop the same size distribution as in the vials with the least competition, but end up approximately 0.10 mg smaller than those in the vials with the least competition. This also suggests that filtering promotes growth better than retention.

4) Vials with the most competition (5 larvae)—These females also have more total food than those in the vials with the least total food (4 larvae), and they grow larger than those females. In this case, the incremental food/larva is more beneficial than the addition of the extra larva is detrimental. Vials with 4 larvae get 8 mg or 16 mg of food, while the vials with 5 larvae get 10 mg or 20 mg of food. The mean Prime and Average female masses for total food levels of 8 mg and 10 mg range from 2.50 mg to 2.52 mg. The masses for total food levels of 16 mg and 20 mg are 1.0 mg higher (40%, 3.45 mg to 3.80 mg). The largest increase is in the vials with 5 larvae and 20 mg total food. There appears to be a change in the growth of female larvae at food levels between 16 mg and 20 mg total food per vial that results in a disproportionate increase in the mass of females. It appears that females in vials with less food switch to retention as they perceive the number of particles decreasing and thereafter grow more slowly, while females in vials with more total food (20 mg per vial and greater) continue to filter and grow at a faster rate, and to a larger size.

5) Vials with the most total food (7 larvae or 8 larvae)—These females grow larger than any other females and take longer to pupate. All these vials have more than 20 mg of food in them. Females in the vials with the least competition grow faster and pupate earlier, so these females delay pupation and become larger. As mentioned earlier, larger filter feeders have an advantage over smaller ones, but as they grow in mass, each increment in mass adds less and less to that advantage. At some point the individual will reach an equilibrium where the filtering is only sufficient to maintain its mass, not increase it. Females in vials with the most total food may grow as fast as those in the vials with the least competition and then extend their larval growth period to increase in size at the expense of their growth rate. For females, large adult mass is more beneficial than early age at pupation.

6) Vials with the most competition (7 larvae or 8 larvae)—These females are smaller than those in any other vials. The total food in these vials ranges from 14 mg to 32 mg (14 mg, 16 mg, 21 mg, 24 mg, 28 mg, 32 mg), yet the females pupate at smaller masses than those with the least total food: 8 mg to 20 mg (8 mg, 10 mg, 12 mg, 15 mg, 16 mg, 20 mg). The total food per vial is higher and the relative food (per larva) is identical, yet the females in the higher density vials do not grow as large. These females must switch from filtering to retaining particles later than those in the vials with the least total food, because they do develop a size distribution that resembles the vials with the least competition and the vials with the most total food. The additional larvae in the vials with the most competition must reduce the available particles sufficiently that the effective food level is lower than in the vials with the lowest total food.

Summary: The environment that the larvae experience in their vials changes over time and the larvae respond depending on the initial conditions of the vials (food level and density). For females, food level (mg/larva) is the most important factor, but competition, total food per vial, and density also influence the mass and age at pupation. Females in the vials with the least competition grow fastest. They pupate at large sizes (not always the largest, but close) and earlier than in the other treatments. These vials are the optimum environment for females within this experiment. These females probably filter particles and pass them rapidly through their guts until they pupate. Pupation probably occurs when the quality of the food particles is insufficient to support further growth. In contrast, females in the vials with the least total food begin retaining food early in larval development. They grow slowly and are among the smallest at pupation. Pupation probably occurs when the quality of the food particles is insufficient to support further growth. Females in vials with 5 larvae (instead of 4 larvae) don’t grow as large at the higher food levels, so the added larva reduces the apparent number of particles and causes a switch to retention before pupation. Retention is less effective than filtering so these females are smaller than those in the vials with the least competition despite the equivalent food/larva. However, females in vials with 5 larvae (instead of 4 larvae) grow larger in the vials with the most competition. In this case the total food per vial is greater and the females switch to retention later than those in the vials with the least total food. They don’t grow as large as the females in the vials with more total food, but they are larger than the ones with the least total food (despite equivalent food/larva). At higher densities (7 or 8 larvae) the females in the vials with the most competition are even smaller at pupation than those in vials with the least total food. These females switch to retention later than those in the vials with the least total food; they develop a distribution of sizes similar to those in the vials with the least competition. Once they switch to retention, the larger number of females reduces the apparent number of particles below the level of that in the vials with the least total food, and the pupae are smaller despite the equivalent food/larva. In these vials, pupation may be triggered by low food particle quantity rather than low quality. Again at high densities (7 or 8 larvae) the females in the vials with the most total food are even larger than those with the least competition. These females filter and pass the food rapidly through their guts until pupation, similarly to those females in the vials with the least competition. However, the large excess of food allows them to continue to grow, albeit at a slower pace, for more than a day after the Prime females in the corresponding vials with least competition have pupated. It is possible that pupation is triggered by larval size rather than by diminished food quality or quantity.
