# Supplementary material for: Competition among Aedes aegypti larvae
Source: PLoS One. 2018 Nov 15;13(11):e0202455. doi: 10.1371/journal.pone.0202455 (PMC6237295; doi:10.1371/journal.pone.0202455)
Supplement: S3 Text — (DOCX) [file pone.0202455.s017.docx]

S3 Text. Competition among males—detailed analysis

Male larvae respond differently to the treatment conditions and interactions than female larvae. While male pupal mass is also affected by the food level more than by density or interactions, density and interactions are relatively more important than food level as compared to females. Food level explains only 41 % to 47 % of the variance in mass at pupation for males, compared to 64% for females. Density explains 12 % to 16 % of the variance in mass at pupation for males compared to 6 % to 8 % for females. The three main interactions explain 27 % to 31 % of the variance for males compared to 20 % to 22 % for females (Tables 2 and 3). These comparisons are qualitatively similar to the MANOVA results in Table 1. Since competition effects show up in the interactions, competition is relatively more significant for males than for females, and more significant for the Average males than for the Prime male. Another difference between males and females is in the way that the Prime male and Average males respond to the interactions. While both Prime and Average females’ masses respond to the interaction treatment combinations in the same way, the Prime male and the Average male masses grow largest in different treatments. The Prime male mass is largest in the treatments with the most total food per vial, similar to the female mass variables, while the Average male mass is largest in the vials with the least competition.

These two experiments look at competition among males in 5 ways: the interactions identify differences in 1) mass and 2) age at pupation under various levels of competition; 3) the difference between the Prime and the Average males indicates possible interference competition; 4) the differences in growth rate of the Prime male in the different treatment combinations is another measure of competition; and 5) the significant regression of the percent of males on the mass of the Prime males at the lower food level.

1 & 2) The Prime male grows to the largest mass and takes the longest to pupate in the vials with the most total food. The Prime male pupates earliest and at the smallest mass in the vials with the most competition. These are both high density treatments. The vials with the least competition produce Prime males that are smaller than the ones with the most total food, and they pupate earlier, however, Prime males in the vials with the least competition have the highest growth rates across all the vials. Prime males in all three interactions grow and pupate like Prime and Average females in the F1 X D3 and F2 X D3 interactions; total food per vial has a positive effect on mass at pupation and competition has a negative effect. However, the effect of the treatments on the age at pupation is different for males and for females; males delay pupation at high total food per vial, like females, but pupate earliest in vials with the most competition, where females pupate earliest in vials with the least competition.

Average males grow largest in the vials with the least competition and are smallest in the vials with the most competition. Another feature of the vials with the least competition is that the Average male mass is greater than the Prime male mass; the male larvae remaining in the vial after the Prime male pupates grow larger than the Prime male on the food resource that is left. Considering that the Prime male outcompetes the other males, when the Prime male pupates, the next larger male should assume the dominant role until he pupates, followed by the next, and so on. At least one of these males must be larger than the Prime male for the Average mass to be greater than the Prime mass. This implies a competitive release after the Prime male pupates as well as a considerable amount of food left over.

Males in the vials with the highest total food per vial take longer and grow larger than the males in the vials with least competition. If optimizing mass against early pupation were the only criteria that male larvae use to determine when to pupate, then the Prime male mass and age at pupation should be the same here as in the vials with the least competition. They are not; males in a high food environment delay pupation and increase further in mass.

3) There is no indication of interference competition based on the size distribution of males at pupation. At the lowest total food per vial this distribution is compressed rather than elongated as it would be if the larval mosquitoes engaged in some kind of interference competition. In vials with higher densities the difference between the Prime male and the Average male mass is approximately the same regardless of the treatment. This smallest difference in size between the Prime male mass and the Average male mass in the vials with the lowest total food per vial may indicate an effect of total food per vial at the lower end of the range as well as at the upper end. The Prime male in these vials is not much larger than those in the vials with the most competition and the age at pupation is also not much greater. However, the Average male mass in these vials is relatively larger than the corresponding masses in the vials with the most competition, resulting in the tighter size distribution. More intense competition for food due to the particle retention by the female larvae reduces the size of the Prime male, but allows the non-Prime males to continue to grow after the Prime male pupates, whereas the most intense competition affects the non-Prime males more.

4) Prime males in the vials with the least competition have the highest growth rates across all the vials. Prime males in the vials with the most competition take less time, and pupate at lower masses than those in the vials with the least competition. They also pupate earlier and at lower masses than Prime males in the vials with the least total food per vial (the same food/larva treatments at the lower density). The effect of increased density is to increase competition despite increasing the total food per vial and maintaining proportional food resources. Under this competitive stress Prime males pupate earlier and at lower masses. The growth rates are also lower, suggesting that the males are smaller not just because they pupate earlier, but because they are not able to grow as well.

5) From the sex ratio experiment we know that at low food levels, the Prime male mass increases as the percent of males in the vial increases. The Average mass does not change so the increase in mass of the Prime male is offset by the decrease in mass of the non-Prime males. The Prime male outcompetes the other males for food (at 3 mg/larva) and the advantage of the Prime male increases as the percent of males increases.

Because the Prime male benefits from the increased percent of males at the low food level, but none of the other mass variables are affected by the percent of males at either food level, the environment that males experience in the vials must take into consideration what the female larvae are doing. During the early growth of the larvae, food levels are expected to appear high because the larvae are small and the quality of the food is initially at its highest. As they grow and females switch from filtering to retention in some vials, the males will experience a reduction in the number of food particles as well. Since the Prime male is affected by the percent of males in exactly the vials where the females are retaining food, it appears that males do not retain food but continue to filter particles and pass them rapidly through their guts even as the particle numbers decrease.

There are only 4 environmental conditions indicated by the three main interactions for males:

1) Vials with the least competition—Prime males grow at the fastest rate and pupate at sizes close to the largest. Average males grow even larger. Since females in these vials also grow at the fastest rate across the experiment, it appears that the Prime male filters particles and grows to a size that allows or triggers pupation, and then the remaining males experience a net increase in food particles that allows them to grow even larger. The Prime male is not retaining particles as females do when particle numbers or quality decreases, but it does sequester some number of particles as they pass through the gut. It is the release of these particles at pupation that drives the non-Prime males to grow further.

2) Vials with the least total food—the Prime male pupates at masses and ages that are almost as low as in the vials with the most competition. In these vials, the females appear to be food limited and switch from filtering to retention earlier than in other vials, reducing the number of particles and the particle quality further. These vials correspond to the conditions in the second experiment where the Prime males benefit from the increased percent of males in the vial. The Prime male competes with the other males for particles, the numbers of which the females affect by retaining the particles in their guts. The low total food per vial causes the females to switch to retention earlier and this causes the males to experience an even lower total food per vial. The Prime male pupates at a size almost as small as in the vials with the most competition and almost as early. As in the vials with the least competition, the non-Prime males experience a small benefit from the additional food made available once the Prime male pupates, and they grow larger than the non-Prime males in the vials with the most competition. While the size distribution of the female larvae is compressed because they switch to a less effective method of feeding at low food levels, the size distribution of the male larvae is compressed because they have less total food available to them due to the retention of the females. The results of the sex ratio experiment indicate that the males are competing in a pure exploitative mode at the low food levels (where one would expect interference competition), the fewer the females present, the more available particles and the larger the Prime male grows. Because the size of the Average males is not affected by the percent of males, the non-Prime males decrease in size proportionately to the Prime male’s increase. This indicates that the males are filtering even at low food levels. If they were retaining food particles as the females appear to do, we would not see this change in the size distribution due to the percent of males in the vials.

3) Vials with the most total food—in these vials, females extend their growth beyond that of females in the vials with the least competition and Prime males do the same thing (both age and mass at pupation are greater). Prime males grow larger and pupate later in these vials than in any others across the experiment. The females filter particles throughout their larval growth and extend that growth period for two days longer than the Prime male. Prime males pupate between 5 and 6 days except in these vials with the most total food. Prime females pupate between 6 and 9 days except in the vials with the highest densities. When food particles are available and/or food quality is still high, the 4th instar larvae of both sexes delay pupation to increase further in size. The Prime male is 0.5 mg to 1.0 mg larger than Prime males in the vials with the least competition.

The Average male in the vials with the most total food are almost as large as the Average males in the vials with the least competition. They are similar in size to the Prime males in the vials with the least competition (.03 mg larger to .05 mg smaller) indicating that males experience little competition in the vials with the most total food. However, they do not experience the release of food particles and grow to be larger than the Prime male as in the vials with the least competition. The most likely explanation for the difference in outcome between the Average males in the vials with the most food and the Average males in the vials with the least competition is that males are constrained or driven to pupate by a certain age so when the Prime male delays pupation it compresses the distribution of ages at pupation for the rest of the males, limiting the benefit that the additional food bestows on the non-Prime males in the vials with the most total food. [The distribution of ages at pupation within microcosms was not analyzed in this experiment.]

4) Vials with the most competition—the Prime male pupates at the earliest age and smallest mass. The Average male mass is also smallest in these vials. Prime and Average female masses are also smallest at the highest Density (7 or 8 larvae/vial) and close to the smallest in the 5 larvae/ vial treatment. The total food per vial is high enough at these high densities so that the larvae filter and grow large enough to develop a size distribution similar to those in the vials with the least competition. As they grow food particles become relatively scarcer and the females switch from filtering to retaining the particles, making them even scarcer. The males respond to the change in the number of food particles by pupating earlier and at the smallest sizes across the experiment.

Summary: The environment that the larvae experience in their vials changes over time and the larvae respond depending on the initial conditions of the vials (food level and density). For males, food level (mg/larva) is also the most important factor, but density, total food per vial and competition (interactions) are relatively more important than for females. Male larvae are also affected by the number and behavior of female larvae. Male larvae filter particles and pass them through their guts, extracting the most available nutrients; they do not appear to change this feeding strategy to retain particles at the expense of their growth rate, as female larvae do. Males in the vials with the least competition grow fastest, and pupate at large sizes, especially the non-Prime males, which pupate at masses larger than the Prime male in each vial. This indicates a release from competition for the non-Prime males when the Prime male pupates. Males in the vials with the least total food experience a reduction in food particles when the females begin retaining food and develop a similar compressed size distribution to those females. They pupate relatively early and at a relatively small size (not the smallest, but close). Males in the vials with the most competition pupate earlier and at smaller sizes than in any other vials; they appear to have less food available to them than those in the vials with the least total food, despite equivalent initial food/larva. The quality and quantity of particles is reduced by the behavior of the female larvae and this causes the males to pupate early and at a small size. In the vials with the most total food, Prime males grow to their largest sizes and delay pupation by a day to attain that size. Average males grow large as well, but not as large as they do in the vials with the least competition. This is possibly because of the delay in pupation by the Prime male; there may be a time constraint on pupation that reduces the benefit from the release of competition observed in the vials with the least competition.
