# Supplementary material for: Competition among Aedes aegypti larvae
Source: PLoS One. 2018 Nov 15;13(11):e0202455. doi: 10.1371/journal.pone.0202455 (PMC6237295; doi:10.1371/journal.pone.0202455)
Supplement: S4 Text — (DOCX) [file pone.0202455.s018.docx]

S4 Text. Competition between males and females—detailed analysis

Female mosquito larvae grow to be larger than male larvae in similar larval environments. Across the experiment, some Prime and Average males are larger than some Prime and Average females, but within any vial, the Prime and Average female masses at pupation are always larger than the corresponding Prime and Average male masses (S5 Table-S8 Table). Comparing the pupal masses across treatment combinations in the interactions shows a larger difference in size between females and males (Prime vs Prime, Average vs Average) in the high food/larva treatments than in the low food/larva treatments. In the F2 X D1 interaction the difference in size parallels the results of the female mass: the largest difference is in the vials with the least competition and the smallest difference is in the vials with the least total food. These treatments are both at the low density (4 larvae/vial). The vials with 5 larvae show intermediate differences between the females and males, with the higher food level also having the greater size difference. In the F1 X D3 and F2 X D3 interactions the greatest differences in size between the females and males are in the high density (7 or 8 larvae) vials with the most total food, and the smallest differences are in the high density (7 or 8 larvae) vials with the most competition. All these differences reflect the mass of females; in vials where the females grow largest, the difference between males and females is largest, and in vials where the females are the smallest, the difference between males and females is also smallest. This shows up in the MANOVA and the ANOVA r squared results (females are affected by food level more than males). Females vary in size according to food level and total food per vial with competition limiting pupal mass at high densities. Males vary less in size than females and they are smaller and pupate earlier. Males also vary in size according to food level and total food per vial, but density and interactions account for 50 % or more of the variance, and competition is important at both low densities and high densities, at least for the non-Prime males. Within the limits of these experiments, females control the availability of food; males, especially the Prime male, escape competition by growing as rapidly as possible and pupating as the available food decreases.

In the F1 X D3 interaction, the difference between Prime females and Prime males in the vials with the least competition is as great as that difference in the vials with the most food. Prime males and females grow at the fastest rate in the vials with the least competition; these vials are expected to be the optimal conditions for the larvae within this experiment. The large difference in size between the Prime female and the Prime male in these vials is the result of optimal growth. Both Prime females and Prime males grow larger in the vials with the most food per vial, but the difference between them remains the same. This suggests that even at very high total food levels there is an optimal largest size for both males and females. Furthermore, the incremental size between Prime females in the vials with the least competition and those in the vials with the most food is only 0.08 mg, while the incremental size between males in the corresponding vials is only 0.10 mg. Prime females delay pupation for 1.53 days to achieve this incremental growth and Prime males delay it for 0.73 days.

Again, in the F1 X D3 interaction, the difference between the Prime females and Prime males in the vials with the least food per vial is as small as that difference in the vials with the most competition. From the second experiment we know that the Prime male benefits from higher percent males under the conditions in the vials with the least food per vial and that the non-Prime males are smaller. The differences between the Prime females and Prime males in these two treatments are smaller than in any other vials across all three interactions. The sizes of the Prime female and Prime male in the vials with the most competition is even lower than their sizes in the vials with the least total food. This suggests that competition in these vials has a greater deleterious effect on growth than a lower total food per vial, which compresses the size distribution of both males and females. In the vials with the most competition, the size distribution is comparable to the other vials at high density; this means that the non-Prime males and non-Prime females are reduced in size by competition more than they are in the vials with the least total food.

If there were interference competition between males and females, the size distributions should be larger at lower food levels rather than smaller as observed. Competition between males and females is exploitative, but females control the food resource in two ways: female larvae grow larger than males and dominate the competition for particles by filtering faster; and female larvae retain food particles when food becomes relatively scarce so that the available particles become even scarcer. The Prime male pupates within 5 to 6 days after hatching except when food availability is high and it extends larval growth to increase in size at the expense of its growth rate. The Average male grows to a size dictated by the initial food level and competition. The Prime female pupates within 6 to 8 days after hatching except when density is high and it extends its larvae growth. Two of these exceptions are at the highest density and lowest food levels (most competition) and two of them are at the highest densities and highest food level (most total food), so there are potentially different causes for the extension in larval growth among females. The Average female mass at pupation reflects the growth patterns of the Prime female.
