# Supplementary material for: Competition among Aedes aegypti larvae
Source: PLoS One. 2018 Nov 15;13(11):e0202455. doi: 10.1371/journal.pone.0202455 (PMC6237295; doi:10.1371/journal.pone.0202455)
Supplement: Supplementary file 20 [file pone.0202455.s019.docx]

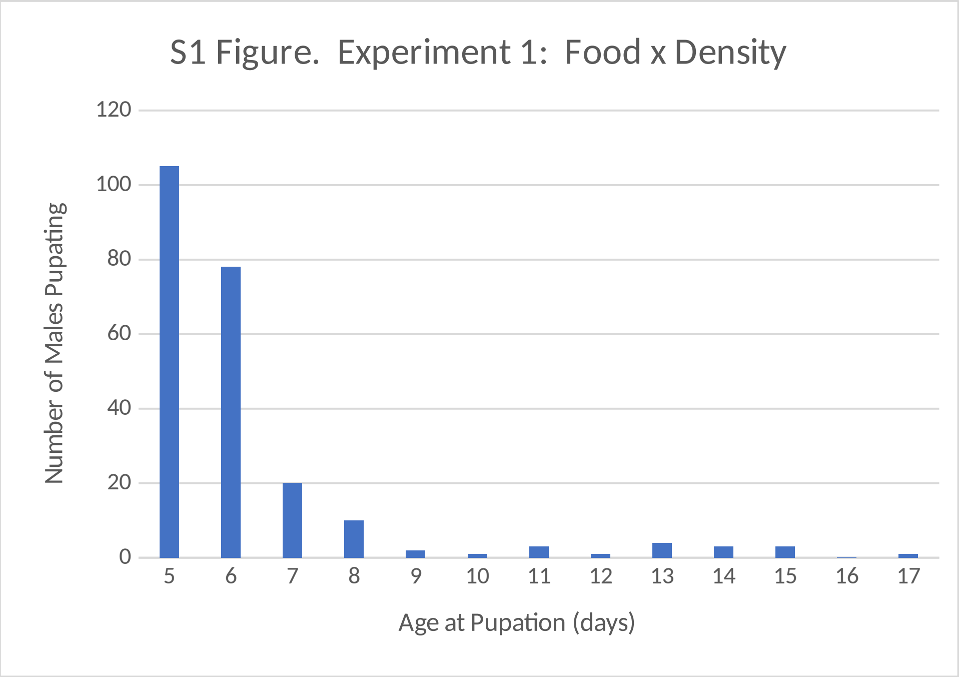

S1 Figure. Experiment 1: Food x Density
120
100
80
60
40
20
0
Number of Males Pupating
5
6
7
8
9
10
11
12
13
14
15
16
17
Age at Pupation (days)
