# Supplementary material for: Competition among Aedes aegypti larvae
Source: PLoS One. 2018 Nov 15;13(11):e0202455. doi: 10.1371/journal.pone.0202455 (PMC6237295; doi:10.1371/journal.pone.0202455)
Supplement: Supplementary file 21 [file pone.0202455.s020.docx]

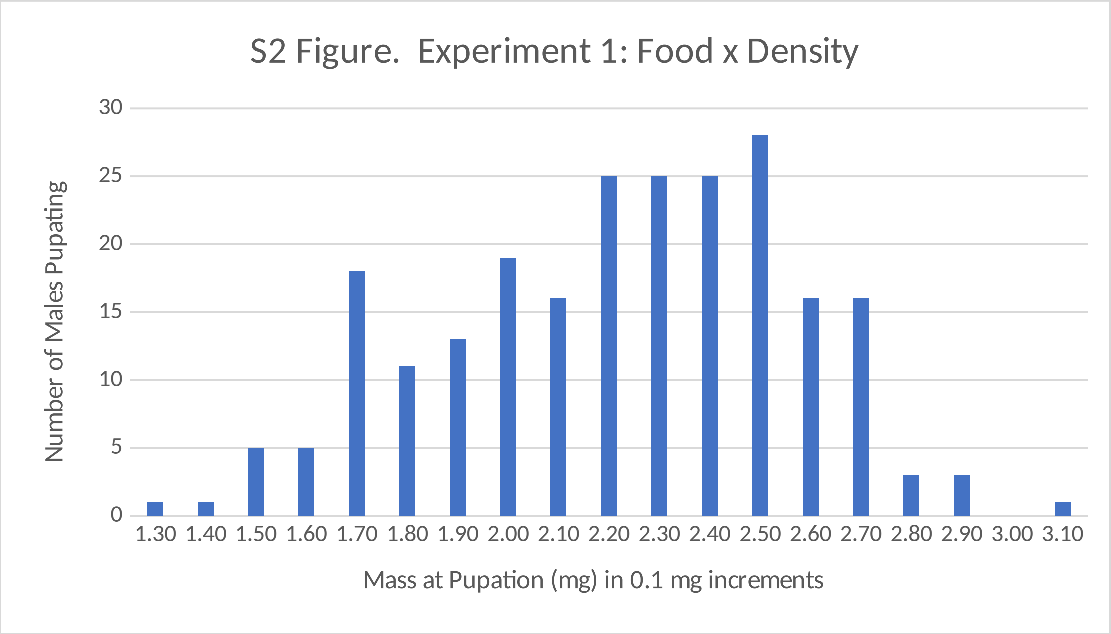
S2 Figure. Experiment 1: Food x Density
Number of Males Pupating
0
5
10
15
20
25
30
1.30
1.40
1.50
1.60
1.70
1.80
1.90
2.00
2.10
2.20
2.30
2.40
2.50
2.60
2.70
2.80
2.90
3.00
3.10
Mass at Pupation (mg) in 0.1 mg increments
