# Supplementary material for: Competition among Aedes aegypti larvae
Source: PLoS One. 2018 Nov 15;13(11):e0202455. doi: 10.1371/journal.pone.0202455 (PMC6237295; doi:10.1371/journal.pone.0202455)

S3 Figure. Experiment 1: Food x Density

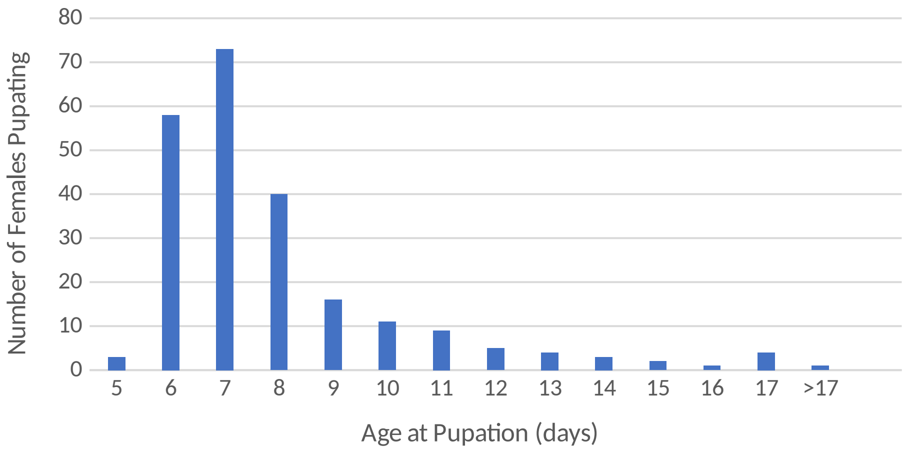

Supplement: Supplementary file 22 [file pone.0202455.s021.docx]
