# Supplementary material for: Competition among Aedes aegypti larvae
Source: PLoS One. 2018 Nov 15;13(11):e0202455. doi: 10.1371/journal.pone.0202455 (PMC6237295; doi:10.1371/journal.pone.0202455)
Supplement: Supplementary file 23 [file pone.0202455.s022.docx]

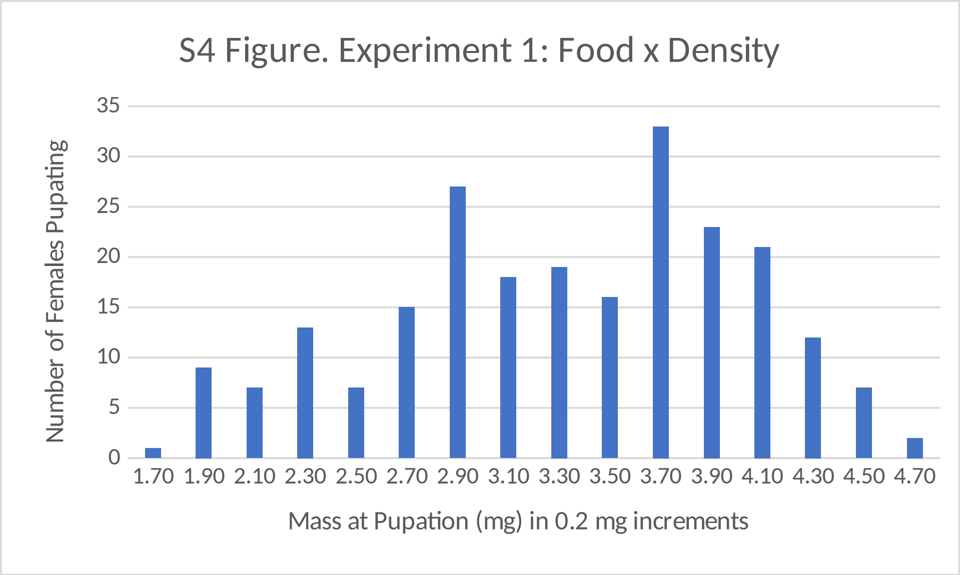
S4 Figure. Experiment 1: Food x Density
Number of Females Pupating
35
30
25
20
15
10
5
0
1.70
1.90
2.10
2.30
2.50
2.70
2.90
3.10
3.30
3.50
3.70
3.90
4.10
4.30
4.50
4.70
Mass at Pupation (mg) in 0.2 mg increments
