# Supplementary material for: Competition among Aedes aegypti larvae
Source: PLoS One. 2018 Nov 15;13(11):e0202455. doi: 10.1371/journal.pone.0202455 (PMC6237295; doi:10.1371/journal.pone.0202455)
Supplement: Supplementary file 24 [file pone.0202455.s023.docx]

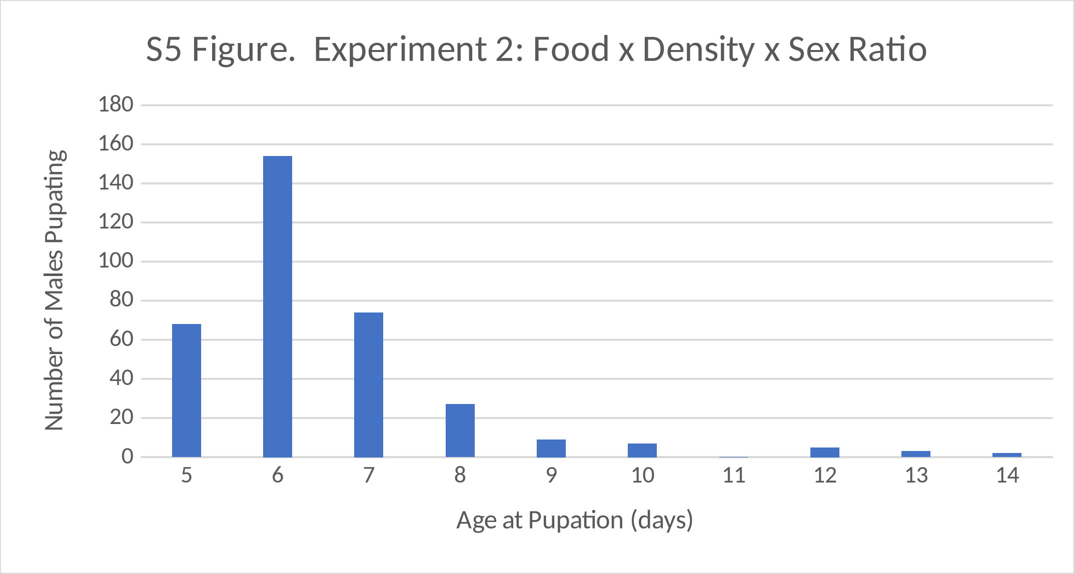
S5 Figure. Experiment 2: Food x Density x Sex Ratio
Number of Males Pupating
180
160
140
120
100
80
60
40
20
0
5
6
7
8
9
10
11
12
13
14
Age at Pupation (days)
