# Supplementary material for: Competition among Aedes aegypti larvae
Source: PLoS One. 2018 Nov 15;13(11):e0202455. doi: 10.1371/journal.pone.0202455 (PMC6237295; doi:10.1371/journal.pone.0202455)
Supplement: Supplementary file 25 [file pone.0202455.s024.docx]

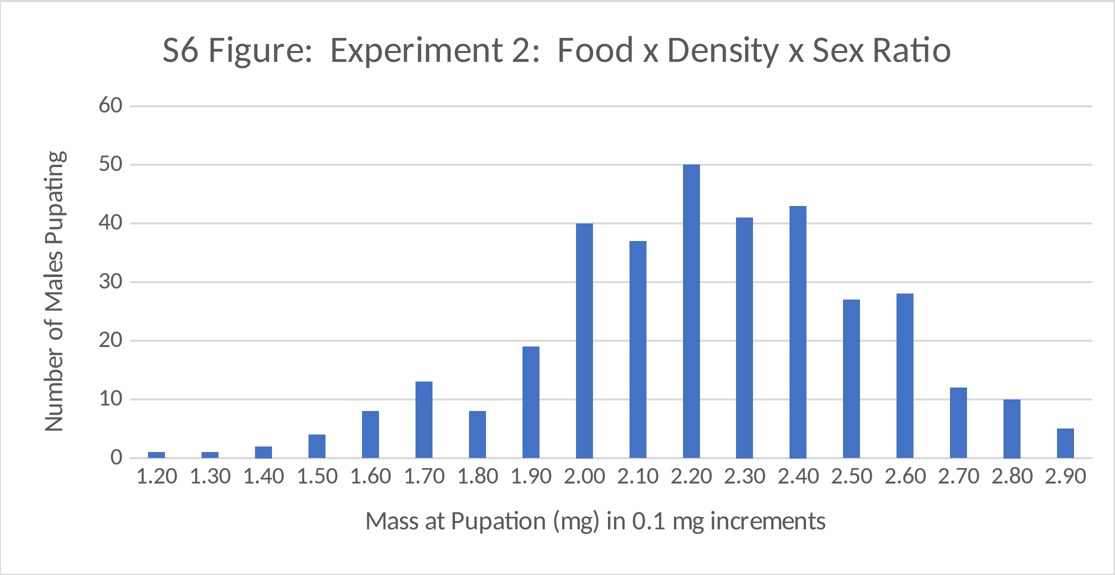
S6 Figure: Experiment 2: Food x Density x Sex Ratio
Number of Males Pupating
60
50
40
30
20
10
0
1.20 1.30 1.40 1.50 1.60 1.70 1.80 1.90 2.00 2.10 2.20 2.30 2.40 2.50 2.60 2.70 2.80 2.90
Mass at Pupation (mg) in 0.1 mg increments
