# Supplementary material for: Competition among Aedes aegypti larvae
Source: PLoS One. 2018 Nov 15;13(11):e0202455. doi: 10.1371/journal.pone.0202455 (PMC6237295; doi:10.1371/journal.pone.0202455)
Supplement: Supplementary file 26 [file pone.0202455.s025.docx]

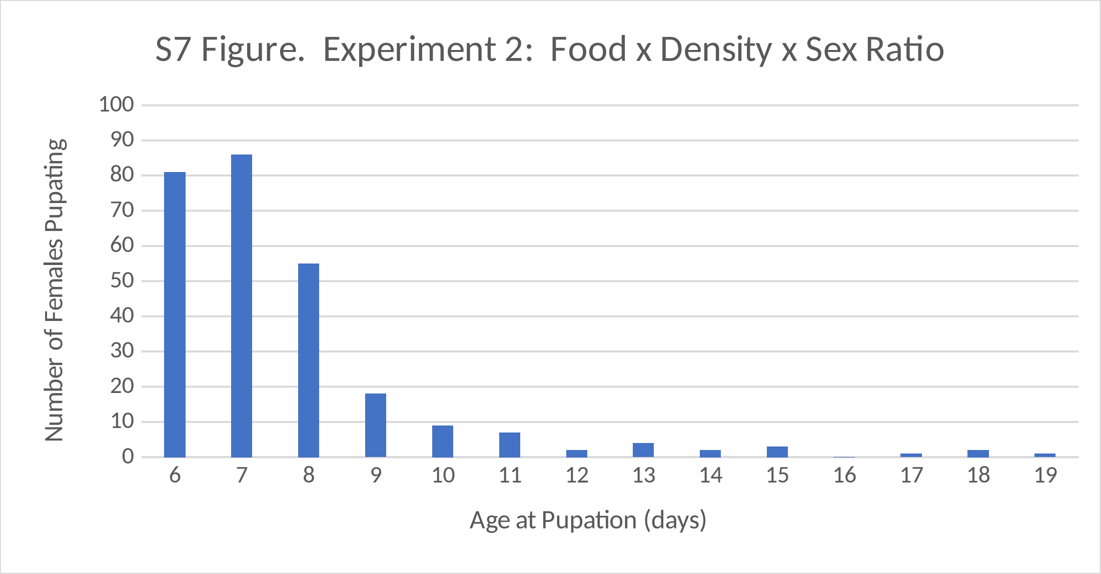
S7 Figure. Experiment 2: Food x Density x Sex Ratio
Number of Females Pupating
100
90
80
70
60
50
40
30
20
10
0
6
7
8
9
10
11
12
13
14
15
16
17
18
19
Age at Pupation (days)
